# Diagnostic performance of microscopic stool examination in *Campylobacter* infection performed by different medical specialties

**DOI:** 10.1002/jgf2.596

**Published:** 2022-11-28

**Authors:** Yumi Hirose, Yusaku Akashi, Yu Sun, Shigeyuki Notake, Atsuo Ueda, Daisuke Kato, Shino Muramatsu, Hiroichi Ishikawa, Hiromichi Suzuki

**Affiliations:** ^1^ Department of General Medicine and Primary Care Tsukuba Medical Center Hospital Tsukuba Japan; ^2^ Division of Infectious Diseases, Department of Medicine Tsukuba Medical Center Hospital Tsukuba Japan; ^3^ Akashi Clinic of Internal Medicine Clinic Osaka Japan; ^4^ Department of Infectious Diseases, Faculty of Medicine University of Tsukuba Tsukuba Japan; ^5^ Graduate School of Comprehensive Human Sciences University of Tsukuba Tsukuba Japan; ^6^ Health Services Research and Development Center University of Tsukuba Tsukuba Japan; ^7^ Department of Clinical Laboratory Tsukuba Medical Center Hospital Tsukuba Japan; ^8^ Research & Development Division, Reagent R&D Department Gosen site, Denka Co., Ltd. Gosen Japan; ^9^ Department of Respiratory Medicine Tsukuba Medical Center Hospital Tsukuba Japan

**Keywords:** antigen testing, *Campylobacter* infection, gastroenteritis, Gram staining, microscopic examination, sensitivity and specificity

## Abstract

**Background:**

Microscopic examination of stool samples can contribute to the early diagnosis of *Campylobacter* gastroenteritis. However, it is unclear whether the diagnostic performance is reliable when performed by physicians.

**Methods:**

This prospective study included fresh stool samples collected from patients with gastroenteritis between August 2018 and March 2020. The samples were used for microscopic examination through Gram staining. Two physicians, a clinical laboratory technician, and microbiologists performed the examinations. In addition, antigen tests (QuickNavi‐Campylobacter; Denka Co., Ltd.) were evaluated for the samples collected between May 2019 and March 2020. Infection with *Campylobacter* spp. was confirmed when stool cultures or polymerase chain reaction tests provided positive results.

**Results:**

Microscopic examination was performed on 205 samples, of which 46 (22.4%) were positive for *Campylobacter* spp. For the microscopic examination, the sensitivity and specificity were 53.5% and 98.1% for physician A, 46.7% and 96.2% for physician B, 63.0% and 100% for the clinical laboratory technician, and 67.4% and 100% for microbiologists, respectively. The antigen testing was evaluated in 131 of the 205 samples and showed a sensitivity of 93.3% and specificity of 99.0%.

**Conclusions:**

Microscopic examination of the stool samples showed high specificity. The sensitivity when the examinations were performed by the physicians was insufficient. The rapid antigen tests can reliably detect *Campylobacter* spp. in stool samples.

## Graphical abstract

Microscopic examination of stool samples demonstrated high specificity. However, the sensitivity was insufficient and varied among examiners depending on their experiences. On the other hand, a rapid antigen tests can reliably detect *Campylobacter* spp. in stool samples.

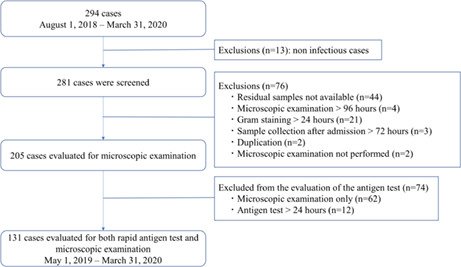

## 1 INTRODUCTION

*Campylobacter* spp. are a common cause of bacterial gastroenteritis,^1^ most of which are self‐limiting.^2^ Nevertheless, early diagnosis is desirable, as early antimicrobial treatment is recommended for severely ill patients and those at risk of serious complications.^1^

Stool culture is generally performed for detecting *Campylobacter* spp. in clinical practice, but it is time‐consuming.^3^, ^4^ Therefore, microscopic examinations of fresh stool samples are often performed concurrently and may contribute to early diagnosis.^5^ Microscopic examinations performed by microbiologists demonstrated modest sensitivity and high specificity compared to stool cultures.^6^, ^7^, ^8^ In Japan, both laboratory technicians and physicians perform microscopic examinations as point‐of‐care testing (POCT), especially in teaching hospitals.^5^, ^9^ However, previous studies have not evaluated its diagnostic performance when conducted by nonmicrobiologists.^6^, ^8^

Another potent POCT is the rapid antigen testing of stool samples but only a few products are commercially available.^3^, ^10^ QuickNavi‐Campylobacter (Denka Co., Ltd.) is a lateral‐flow‐type antigen testing with a sensitivity of 75.6% and specificity of 98.6% in patients with enteritis.^10^ Nevertheless, the evidence on its validity in diagnostic performance has been insufficient, warranting further research to evaluate its clinical utility.

This study aimed to assess the diagnostic performance of microscopic examinations with Gram staining and to compare the results when performed by physicians and microbiologists. In addition, we compared the diagnostic performance of microscopic examination with that of rapid antigen testing using the same stool samples. We employed both stool culture and polymerase chain reaction (PCR) as references.

## 2 METHODS

### 2.1 Study design and setting

This observational study was conducted at Tsukuba Medical Center Hospital (TMCH) between August 1, 2018 and March 31, 2020. This study first evaluated the clinical performance of direct microscopic examination (August 1, 2018 to April 30, 2019) and conducted the evaluation of antigen testing in addition to the direct microscopic examination from May 1, 2019, onwards.

We prospectively enrolled fresh stool samples that were provided for stool culture in daily clinical practice. Patients were asked to self‐collect stool samples in plastic containers that were immediately sent to a microbiological laboratory at TMCH, and Gram staining and antigen testing were performed within 24 h. Direct microscopic examinations were performed for Gram‐stained samples within 96 h.

The current observational study used an opt‐out consent procedure and was approved by the ethics committee of TMCH (approval number: 2021‐020). Each patient data was anonymized before conducting data analysis.

### 2.2 Patient enrolment and data collection

This study included outpatients and inpatients (within 72 h of hospitalization) suspected of having infectious gastroenteritis. The exclusion criteria were (i) patients who did not suffer from infectious gastroenteritis, (ii) where residual samples were not available, (iii) patients who opted out of participation in the study, and (iv) duplicate cases.

We collected the patients' clinical information retrospectively from their medical records. The collected clinical information included age, sex, comorbidities, symptoms (fever, chills, diarrhea, hematochezia, vomiting, abdominal pain, and myalgia/arthralgia), and preceding antibiotic use.

### 2.3 Procedures for microscopic examinations and antigen testing

Both direct microscopic examination and antigen testing utilized fresh stool samples within 24 h of sample submission. For microscopic examination, smeared samples were Gram‐stained with Bartholomew and Mittwer‐modified reagents (Muto Pure Chemicals Co. Ltd.). The smeared samples were anonymized by study numbers, kept in a storage box, and thereafter were observed at 1000× magnification. Microscopic examinations were performed by two physicians (Physician A [Board certified family medicine and geriatric medicine physician] and Physician B [Board‐certified emergency physician] with 16‐year and 10‐year physician experience, respectively, as at the study initiation), a clinical laboratory technician, and microbiologists. We independently checked and recorded the presence or absence of spiral‐shaped Gram‐negative rods. Clinical information had not been reviewed at the time of microscopic examination.

Antigen testing was performed in accordance with the manufacturer's instructions. In brief, samples were dispensed into the extraction solution and added into the kit wells. Similar to other lateral flow antigen tests, the test was considered positive for *Campylobacter* spp. if a positive test line appeared.

### 2.4 Procedures for stool culture

Stool samples were incubated micro‐aerobically on Campylobacter agar with 5% antimicrobics and 10% sheep blood (Becton, Dickinson and Company, Ltd.) using GasPack method for 48 h at 35°C. After 48‐h incubation, translucent drop‐like colonies were selected and examined microscopically. *Campylobacter* spp. were considered positive on stool culture when microscopic examination of the colonies revealed spiral‐curved Gram‐negative rods.

### 2.5 Procedures for PCR

After being used for microscopic examination, antigen testing, and culture, the residual stool samples were stored at −80°C. The samples were sent to Denka Co. Ltd. for PCR examination if the stool cultures were negative for *Campylobacter* spp.

Frozen stool samples were thawed, and DNA was extracted and purified using the QIAGEN QIAamp DNA Stool Kit (QIAGEN. N.V.). The extracted samples were subjected to real‐time PCR using the CycleavePCR *Campylobacter* (*jejuni/coli*) Typing Kit (Takara Bio Inc.). The PCR was performed following the manufacturer's instructions: initial denaturation at 95°C for 3 s, followed by 45 cycles of 95°C for 5 s, 55°C for 10 s, and 72°C for 20 s.

As an additional analysis, a multiplex PCR system, FilmArray Gastrointestinal (GI) Panel (Biofire Diagnostics, Inc.), was used if samples tested positive for the antigen testing but negative for the culture and PCR examinations.

### 2.6 Statistical analyses

The sensitivity and specificity of microscopic examination and antigen testing were calculated based on the combined results of stool culture and PCR. The 95% confidence intervals were determined by using Clopper and Pearson intervals. In addition, we divided the study period into early and late periods according to the order of sample submission, where each study period contained the same sample size. The sensitivity and specificity of microscopic examinations were compared between the periods. Cohen's and Fleiss' Kappa indices were determined to evaluate the agreement of the microscopic examinations between different examiners.

Categorical variables were compared using Fisher's exact test. *p* Values <0.05 were considered statistically significant. Variables of clinical symptoms that were statistically significant in the univariate analysis were included in a multivariate logistic regression analysis. Cases with missing data were omitted from the analyses. All statistical analyses were conducted using R version 4.1.2. (R Foundation for Statistical Computing).

## 3 RESULTS

**FIGURE 1:**
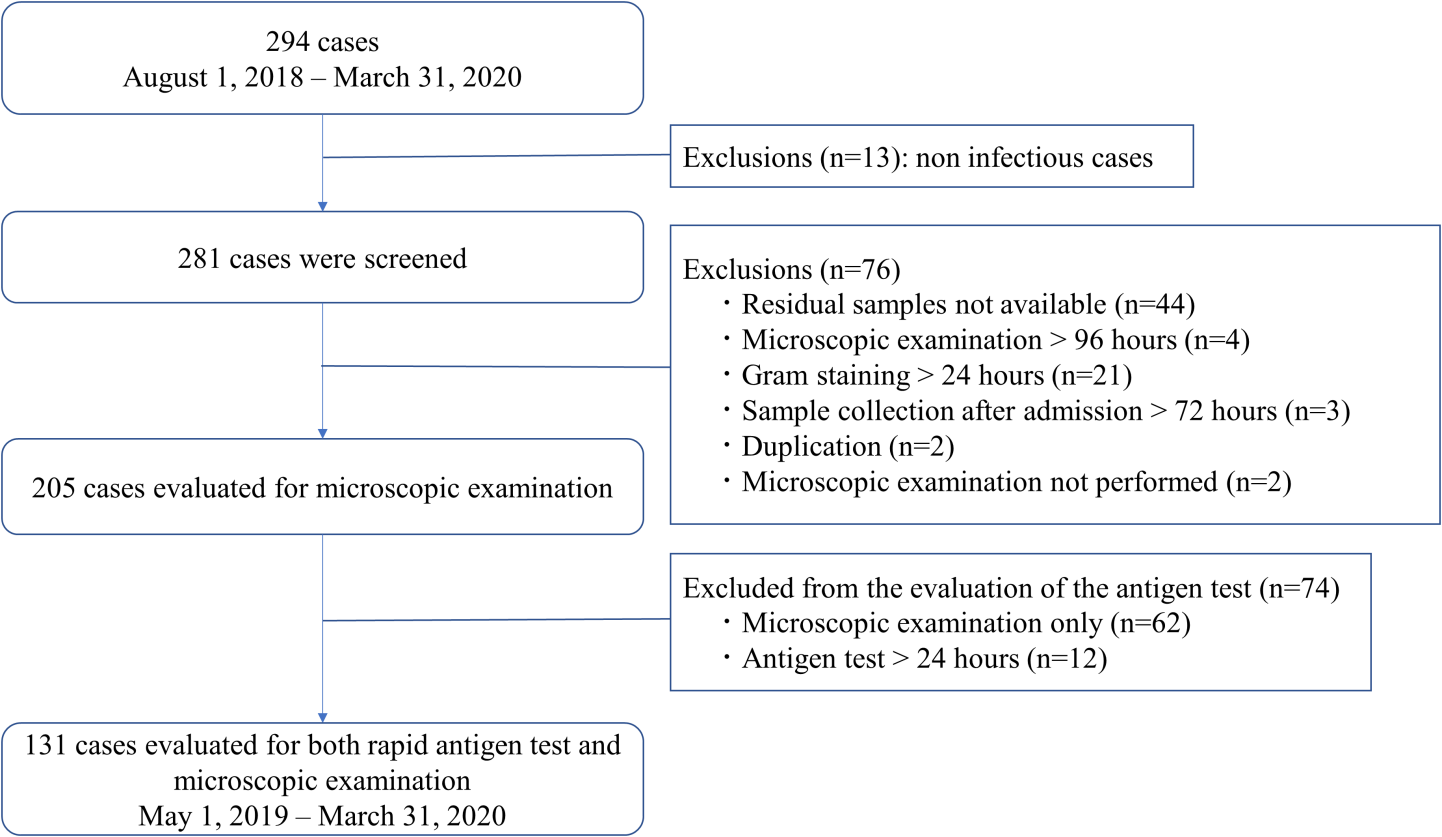
Flowchart showing patient selection

Two hundred and ninety‐four submitted stool culture specimens were consecutively included. Of these, 13 patients who were determined to have no apparent gastroenteritis (e.g., based on screening on admission) were removed, and 281 patients were screened in this study. Of these 281, we excluded 76 patients: 44 for unavailability of residual samples; four for Gram staining to microscopic examination >96 h; 21 for Gram staining >24 h; three for sample collection >72 h after admission; two for duplication; two for not undergoing a microscopic examination. Finally, we included 205 patients for the evaluation of direct microscopic examinations (Figure 1). Stool culture was positive for *Campylobacter* spp. in 44 patients, and PCR testing provided additional two positive patients; thus, 46 (22.4%) were considered positive for *Campylobacter* spp.

### 3.1 Demographic data of included patients

Patient demographic data are summarized in Table 1. The median age was 23.0 years (interquartile range [IQR]:8.0–40.0), and 94 (45.9%) of patients were female. Comorbidities existed in 23 (11.2%) patients and included seven (3.4%) patients with cerebrovascular disease, six (2.9%) with diabetes mellitus, and four (2.0%) with dementia. Prior to the sample collection, antibiotics had been prescribed for 27 (13.2%) patients.

**TABLE 1:** Demographic data of the study population and cases identified with *Campylobacter* infection.

Variable	*Campylobacter* spp.			*p*‐Value
	Total	Positive	Negative	
	*n* = 205 (100%)	*n* = 46 (22.4%)	*n* = 159 (77.6%)	
Age [IQR]	23.0 [8.0, 40.0]	24.0 [20.3, 28.8]	23.0 [6.0, 44.0]	0.77
Sex (Female) (%)	94 (45.9)	23 (50.0)	71 (44.7)	0.61
Comorbidities (%)	23 (11.2)	2 (4.3)	21 (13.1)	0.50
Immunosuppressive state	2 (1.0)	1 (2.2)	1 (0.6)	—
Rheumatic disease	2 (1.0)	1 (2.2)	1 (0.6)	—
Any malignancy	4 (2.0)	0 (0.0)	4 (2.5)	—
Diabetes mellitus	6 (2.9)	0 (0.0)	6 (3.8)	—
Cerebrovascular disease	7 (3.4)	0 (0.0)	7 (4.4)	—
Dementia	4 (2.0)	0 (0.0)	4 (2.5)	—
Myocardial infarction	1 (0.5)	0 (0.0)	1 (0.6)	—
Congestive heart failure	2 (1.0)	0 (0.0)	2 (1.3)	—
Chronic pulmonary disease	1 (0.5)	0 (0.0)	1 (0.6)	—
Liver cirrhosis	1 (0.5)	0 (0.0)	1 (0.6)	—
Prior antimicrobial therapy (%)	27 (13.2)	6 (13.0)	21 (13.2)	1
Bristol scale (median [IQR])	5.0 [5.0, 6.0]	6.0 [5.0, 7.0]	5.0 [4.0, 6.0]	0.002
Bristol ≧6 (%)	97 (47.3)	30 (65.2)	67 (42.1)	0.007
Symptoms (%)				
Abdominal pain	128 (62.4)	43 (93.5)	85 (53.5)	<0.001
Vomiting	74 (36.1)	5 (10.9)	69 (43.4)	<0.001
Diarrhea	171 (83.4)	45 (97.8)	126 (79.2)	0.001
Hematochezia	54 (34.2)	12 (28.6)	42 (36.2)	0.45
Fever (>37.8°C)	78 (38.0)	32 (69.6)	46 (28.9)	<0.001
Myalgia or arthralgia	19 (9.3)	8 (17.4)	11 (6.9)	0.04
Chills	17 (8.3)	13 (28.3)	4 (2.5)	<0.001

Abbreviation: IQR, interquartile range.

### 3.2 Clinical characteristics of patients infected with *Campylobacter* spp.

Patients with *Campylobacter* spp. infections were more likely to have diarrhea (97.8% vs. 79.2%, *p* = 0.001), abdominal pain (93.5% vs. 53.5%, *p <* 0.001), fever (69.6% vs. 28.9%, *p* < 0.001), and chills (28.3% vs. 2.5%, *p* < 0.001) than non‐infected patients, but vomiting was less frequent (10.9% vs. 43.4%, *p* < 0.001; Table 1).

Multivariate analysis revealed that abdominal pain, vomiting, fever, and chills were significantly associated with *Campylobacter* infections (Table 2). The multivariate odds ratios (OR) for each variable were as follows: abdominal pain, OR 13.7 (95% CI: 3.65–51.7); vomiting, OR 0.18 (95% CI: 0.06–0.55); fever, OR 6.14 (95% CI: 2.43–15.5); and chills, OR 6.50 (95% CI: 1.23–34.3).

**TABLE 2:** Odds ratios (with 95% confidence intervals) of clinical symptoms for *Campylobacter* infection.

Symptoms	Crude OR (95%CI)	*p*‐Value	Adjusted OR (95%CI)	*p*‐Value
Abdominal pain	12.4 (3.71–64.9)	<0.001	13.7 (3.65–51.7)	<0.001
Vomiting	0.16 (0.05–0.44)	<0.001	0.18 (0.06–0.55)	0.002
Diarrhea	11.7 (1.84–488.9)	0.001	8.31 (0.98–70.76)	0.05
Hematochezia	0.71 (0.30–1.60)	0.45	—	—
Fever (>37.8°C)	5.56 (2.61–12.4)	<0.001	6.14 (2.43–15.5)	<0.001
Myalgia or arthralgia	2.81 (0.92–8.32)	0.04	0.61 (0.14–2.7)	0.52
Chills	15.0 (4.28–67.1)	<0.001	6.50 (1.23–34.3)	0.03

*Note*: Variables of *p* < 0.05 in univariate analyses were included in the multivariate analysis. The 95% confidence intervals are shown in parentheses.

Abbreviations: CI, confidence interval; OR, odds ratio.

### 3.3 Diagnostic performance of microscopic examinations

The results of the microscopic examination are described in Table 3. Microbiologists showed the highest sensitivity of 67.4% and specificity of 100% for detection of *Campylobacter* spp. Although the sensitivity was 53.5% for physician A and 46.7% for physician B, the specificities exceeded 90% for all examiners.

**TABLE 3:** Diagnostic performance of microscopic examinations by each examiner and rapid antigen test

Examiner/test	Sensitivity, % (95% CI)	Specificity, % (95% CI)	PPV, % (95% CI)	NPV, % (95% CI)
Physician A	53.5 (37.7–68.8)	98.1 (94.4–99.6)	88.5 (69.8–97.6)	88.3 (82.5–92.7)
Physician B	46.7 (31.7–62.1)	96.2 (91.9–98.6)	77.8 (57.7–91.4)	86.2 (80.2–91.0)
Clinical laboratory technician	63.0 (47.5–76.8)	100 (97.7–100)	100 (88.1–100)	90.2 (84.8–94.2)
Microbiologists	67.4 (52.0–80.5)	100 (97.7–100)	100 (88.8–100)	91.4 (86.2–95.1)
Rapid antigen test^a^	93.3 (77.9–99.2)	99.0 (94.6–100)	96.6 (82.2–99.9)	98.1 (93.2–99.8)

*Note*: Sensitivity, specificity, positive predictive value, and negative predictive value are provided with 95% confidence intervals.

Abbreviations: CI, confidence interval; NPV, negative predictive value; PPV, positive predictive value.

^a^ The diagnostic performances of rapid antigen test were evaluated using a subpopulation of 131 cases.

All examiners demonstrated an increased sensitivity during the later study period (Table 4). The sensitivities in the early (*n* = 102) and late (*n* = 103) study periods were 50.0% and 57.9% for the physician A, 34.6% and 63.2% for the physician B, 59.3% and 68.4% for the laboratory technician, and 63.0% and 73.7% for microbiologists, respectively.

**TABLE 4:** Diagnostic performances of microscopic examinations in the early and late period of the study.^a^

	Sensitivity, % (95% CI)		Specificity, % (95% CI)	
Examiner	Early study period	Late study period	Early study period	Late study period
Physician A	50.0 (29.1–70.9)	57.9 (33.5–79.7)	100 (95.0–100)	96.3 (89.7–99.2)
Physician B	34.6 (17.2–55.7)	63.2 (38.4–83.7)	97.3 (90.6–99.7)	95.1 (88.0–98.7)
Clinical laboratory technician	59.3 (38.8–77.6)	68.4 (43.4–87.4)	100 (95.2–100)	100 (95.7–100)
Microbiologists	63.0 (42.4–80.6)	73.7 (48.8–90.9)	100 (95.2–100)	100 (95.7–100)

*Note*: Each study period would contain the same sample size (early, *n* = 102; late, *n* = 103).

Abbreviation: CI, confidence interval.

^a^ The study period was divided into early and late periods according to the order of sample submission.

The Fleiss' kappa value among all examiners for all samples was 0.68, while Cohen's kappa ranged from 0.51 to 0.89 (Figure S1). The agreement between the clinical laboratory technician and microbiologists was the highest, and that between the two physicians was the lowest.

### 3.4 Diagnostic performance of antigen testing

Stool samples from 131 patients including 30 positive cases were subjected to both antigen testing and microscopic examination. The rapid antigen testing showed a sensitivity of 93.3% and specificity of 99.0% (Table 3).

There were two false negative, one false positive, and one invalid results. For the sample with a false positive result, an additional FilmArray assay detected *Campylobacter* spp. The two stool samples with false negative results did not contain solid pieces (Bristol scale 7) and grew a few colonies of *Campylobacter* spp. on the medium.

When evaluating the same stool samples, the sensitivity of antigen testing was higher than that of microscopic examination by all examiners (Table S1).

## 4 DISCUSSION

This prospective study showed that the sensitivity of microscopic examinations was not sufficient for detecting *Campylobacter* spp., regardless of the examiner. The sensitivity was low especially when physicians performed the examination, although the specificity of all examiners exceeded 90%. Compared with microscopic examinations, the rapid antigen test showed higher diagnostic performance.

Our study revealed the test performance of the microscopic examination was comparable with previous studies which reported the sensitivities of 43.5%–65.5% and specificities of 88.0%–99.4%.^6^, ^8^, ^11^ Microscopic examinations performed by physicians were less sensitive than those performed by laboratory technician and microbiologists. In addition, the laboratory technician and microbiologists showed high agreement (Cohen's kappa = 0.90), whereas physicians showed the least agreement (Cohen's kappa = 0.511).^12^ Physicians do not regularly perform microscopic examinations. Wang et al.^7^ reported that the inexperience of a microscopist may cause false negative test results. In this study, all examiners' sensitivity improved in the later period compared to that in the early period. Even for inexperienced physicians, the sensitivity of the examination can be improved through experience and training.

*Campylobacter* infection was significantly associated with abdominal pain, vomiting, fever, and chills (Table 2). Consistent with our study, previous studies reported that vomiting was less frequent in *Campylobacter* infection,^13^ while fever and diarrhea were more common^3^, ^8^. Gillespie et al.^14^ showed that vomiting was more likely to occur in younger children, although no association between vomiting and age was observed in this study. Vomiting usually occurs in toxin‐producing (e.g., *Staphylococcus aureus* and *Bacillus cereus*) and small‐intestinal types of infectious gastroenteritis. C*ampylobacter* spp. generally affects the large intestine, which may result in a lower frequency of vomiting or nausea. Another useful clinical manifestation is the presence of polymorphonuclear leukocytes (PMNL) in stools. The presence of PMNL indicates an inflammation of the bowels in *Campylobacter* gastroenteritis^15^; however, it was not examined in our study. These clinical features may help to suspect *Campylobacter* spp. as the causative microorganism of acute gastroenteritis.

The rapid antigen test, QuickNavi‐Campylobacter, demonstrated favorable sensitivity and specificity results. The sensitivity of the antigen test was better than that of microscopic examination for the same stool samples. Regarding the two samples with false negative results in the antigen test, a small number of colonies were detected in culture. We believe that the low bacterial load in the samples led to false negative results. As for the specificity, one false positive case was confirmed to be *Campylobacter* spp. positive by further evaluation using the FilmArray assay.^16^ In this study, the antigen testing gave positive results only when *Campylobacter* spp. truly existed in samples. In addition to favorable diagnostic performance, antigen testing can provide results in a short time, which may help early diagnosis and proper management in patients with gastroenteritis.

Several multiplex molecular assays have been developed and used in clinical settings for other types of POCT. These systems can detect multiple pathogens with high sensitivity^16^, ^17^ and are beneficial for vulnerable individuals, such as hospitalized or immunocompromised patients.^18^

Appropriate use of each type of POCT is crucial, in different clinical settings. Microscopic examinations and antigen testing can be performed in primary care facilities due to the short turnaround time, easy procedure, and the unnecessity of special equipment. Nevertheless, our study indicated that microscopic examinations by physicians could not reliably rule out *Campylobacter* infection. Antigen testing showed higher sensitivity than microscopic examinations, and the interpretation of the test results can be more objective, although evaluated only in a small study population^10^, ^19^ and not commercially available. Multiplex molecular assays have demonstrated excellent diagnostic performance^16^, ^17^; however, the requirement of special equipment and expensive examination cost can limit their clinical usefulness. Given that most patients with gastroenteritis suffer mild illnesses in the primary care settings, the use of multiplex molecular assays should be reserved for hospitalized or emergency department patients.

The microscopic examinations can contribute to antimicrobial stewardship.^20^ An appropriate choice of empiric antimicrobials depends on the assumption of the causative microorganisms. The ability to assume the causative microorganisms can be achieved by microscopic examinations.^20^ In addition, the pedagogical effect regarding infectious diseases can be expected when physicians can successfully associate the results of the microscopic examinations with the treatment strategy.^20^

The competency for microscopic examinations can be enhanced through lectures, group discussions, and hands‐on workshops. Among them, hands‐on workshops showed the highest education effect.^21^ Our study also indicated that the experience acquired by performing microscopic examinations contributed to improving its sensitivity. Besides, an appropriate feedback increases the efficacy of the educational process.^22^ The practitioner can be effectively trained by a well‐experienced examiner for microscopic examinations, for example, those with a license in performing microbiological examinations and those with a sensitivity of over 60% in performing microscopic examinations.^6^, ^8^, ^11^ To maximize the educational effect, we suggest that the residency training program should start with hands‐on workshops during orientation and that the residents should perform microscopic examination while receiving continual feedback. Regular conferences, focused on microscopic examination, are also effective in improving competency.

This study had some limitations. First, we used frozen samples for the PCR examination. Therefore, the frozen storage process may have affected the PCR results.^23^ Second, the stool culture and PCR methods used in this study could not distinguish between the species of *Campylobacter*. The examination results and clinical manifestations may possibly differ among *Campylobacter* species. Third, a few examiners performed microscopic examinations, and data on patients' symptoms were not prospectively collected from their medical records. Therefore, further studies are needed to validate the generalizability of our results.

## 5 CONCLUSIONS

Microscopic examination of stool samples demonstrated high specificity when using the combined results of culture and PCR as references. However, the sensitivity was insufficient when the examinations were performed by the physicians. Microscopic examinations by well‐trained examiners and the use of rapid detection tests can reliably detect *Campylobacter* spp.

## AUTHOR CONTRIBUTIONS

Y. H. collected data, produced figures, and drafted the manuscript. Y. A. planned the study, collected data, constructed the database, and analyzed the data. D. K. performed the PCR analysis for *Campylobacter* spp. H. S. supervised the study. All the authors contributed to the preparation of the manuscript and met the criteria endorsed by the International Committee of Medical Journal Editors (ICMJE).

## CONFLICT OF INTEREST

Denka Co., Ltd. provided free antigen test kits for this study and performed the PCR examinations. H.S. received lecture fees from Denka. Co. Ltd. D. K. and S. M. belong to Denka Co., Ltd.

## ETHICAL APPROVAL AND PATIENT CONSENT

This study used an opt‐out consent procedure and was approved by the ethics committee of TMCH (approval number: 2021‐020).

## CLINICAL TRIAL REGISTRATION

The study protocol was registered on UMIN‐CTR (UMIN000038341).

## Supporting information


Figure S1.

Table S1.
