# Supplementary figures and images for: Diagnostic performance of microscopic stool examination in Campylobacter infection performed by different medical specialties
Source: J Gen Fam Med. 2022 Nov 28;24(2):102–9. doi: 10.1002/jgf2.596 (PMC10000277; doi:10.1002/jgf2.596)

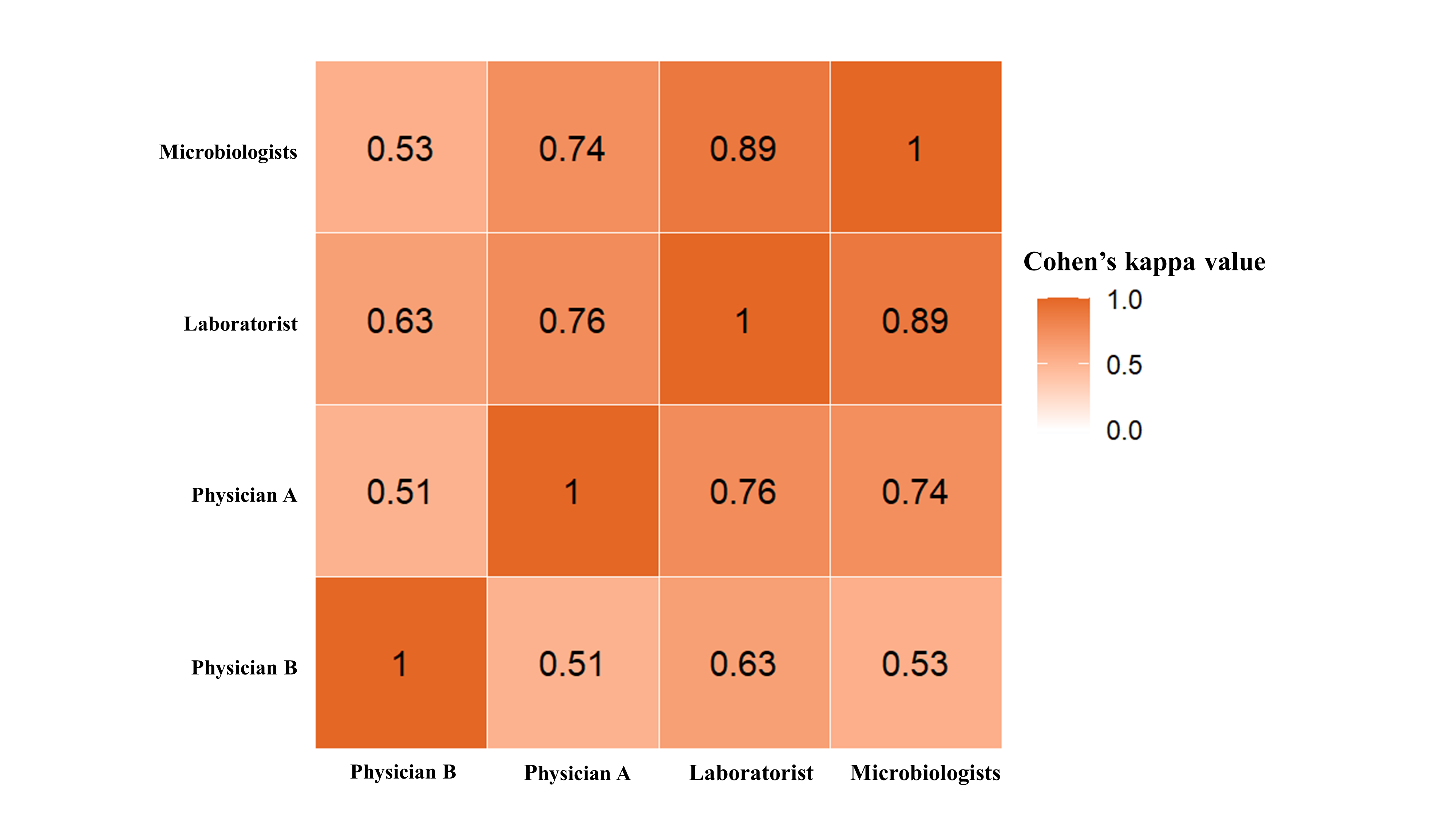

Supplement: Supplementary file 1 — Figure S1. [file JGF2-24-102-s002.TIF]
